# Evaluation of the association between variants of the ZNF536 transcription factor gene, a family history of alcoholism, and the severity of schizophrenia

**DOI:** 10.1192/j.eurpsy.2026.11268

**Published:** 2026-07-31

**Authors:** V. Golimbet, T. V. Lezheiko, A. O. Kurishev

**Affiliations:** Clinical Genetics Laboratory, Mental Health Research Center, Moscow, Russian Federation

## Abstract

**Introduction:**

According to genome-wide analyses, the ZNF536 transcription factor gene is associated with some mental illnesses, including schizophrenia. (Benito E. Et al. 2015, Karpov D.S. et al. 2024). Model studies have shown that mutations in ZNF536 are associated with stress and its consequences (Kaufman J. et al. 2018).

**Objectives:**

To assess the association between ZNF536 rs2053079(A/G), rs8104557(T/C), rs7259428(T/C), rs7249968(T/C), rs334229(A/G) polymorphisms and their haplotypes, childhood psychological trauma (family history of alcoholism) and the severity of schizophrenia assessed by the severity of symptoms.

**Methods:**

A sample of 663 patients with schizophrenia (426 women and 237 men) was divided into 2 groups - with a family history of alcoholism (n=162) and without it (n=501). Statistical processing of the results was performed in Statistica (version 10) using the Pearson Chi2 criterion for qualitative features. To identify haplotypes for polymorphisms rs2053079(A/G), rs8104557(T/C), rs7259428(T/C), rs7249968(T/C), rs334229(A/G) and analyze their association with a family history of alcoholism and the severity of schizophrenia symptoms, the statistical online program SNPStats (https://www.snpstats.net) was used.

**Results:**

An association of two polymorphic loci rs2053079, rs8104557 with the severity of general psychopathological symptoms is found in a group of patients with a family history of alcoholism. Patients with the GG (rs2053079(A/G)) and CC (rs8104557(T/C)) genotypes have the highest symptom severity compared to other genotype*environment combinations. The GCTG haplotype is associated with a family history of alcoholism, which contained risk alleles for the rs2053079(A/G), rs8104557(T/C) and rs7259428(T/C) loci.

**Conclusions:**

The results expand our understanding of the association of ZNF536 variants with schizophrenia, in particular, indicating the contribution of these variants to the phenotype associated with the severity of its course in patients with childhood psychological trauma.

**Disclosure of Interest:**

None Declared

